# Effects of substrate bias on the sputtering of high density (111)-nanotwinned Cu films on SiC chips

**DOI:** 10.1038/s41598-022-19825-x

**Published:** 2022-09-14

**Authors:** Zi-Hong Yang, Po-Ching Wu, Tung-Han Chuang

**Affiliations:** Institute of Materials Science and Engineering, National Taiwan University, Taipei, 106 Taiwan

**Keywords:** Engineering, Materials science

## Abstract

This article presents a study of the influence of the substrate bias on the microstructure, preferred orientation, and mechanical and electrical properties of nanotwinned Cu film. The formation of a nanotwinned structure and (111) surface orientation can be properly controlled by applied substrate bias. High density nanotwinned structures were introduced into Cu films sputtered on SiC substrates with over 90% of (111)-orientation at − 150 V. Densely packed Cu nanotwins were observed within the columnar grains stacked up on each other along the film growth direction, with an average twin spacing of 19.4 nm. The Cu films deposited on SiC substrate via bias sputtering had surface roughness of 8.6 to 15.8 nm. The resistivity of the copper nanotwinned films sputtered with various substrate biases varied. The optimal indentation, 2.3 GPa, was found in the nanotwinned Cu film sputtered with a bias voltage of − 150 V. The effects of Ar ion bombardment on microstructure, surface morphology and properties are further discussed.

## Introduction

Today, the current trend for advanced electronic applications is to develop high frequency, high temperature, high efficiency, and even high radiation resistance during operation^1,2^. Hence, silicon (Si) is ceding its dominance in power devices to wide-bandgap semiconductors such as silicon carbide (SiC), for this material offers a viable and real alternative for proper device performance in such extreme environments^3^. Due to its extraordinary physical and electrical properties, SiC is currently under intense development and has become a particularly interesting challenger to traditional Si for opportunities to produce dedicated devices and integrated circuits (ICs) able to work under extreme conditions.

It is known that the backside metallization of power devices has a great impact on their subsequent application, such as heat dissipation and die attachment. Pure metals with high conductivity are usually employed. However, in their pure, unalloyed states, many metals are too soft, susceptible to corrosion damage, and expensive. Several metallurgical methods, such as grain refinement, solid solution alloying and cold working, can improve the strength of metallic materials. These strengthening approaches entail the introduction of various types of defects, including grain boundaries, reinforcing phases and dislocations. Unfortunately, the presence of these defects can cause scattering of conducting electrons and reduce the electrical conductivity. Therefore, achieving an excellent combination of mechanical properties and electrical conductivity in conductive materials is highly desirable.

Coherent twin boundaries (CTBs) are often thought to be inherently resistant to dislocation slip and to exhibit electrical resistivity of about one order lower than that of high-angle grain boundaries. Hence, a Cu structure having a high density of nanotwins seems to be an appropriate solution due to its excellent mechanical properties^4–6^, good conductivity^7,8^, proper electromigration resistance and superior thermal stability^9–13^. Previous studies have proved that the nanotwinned structure in copper can enhance the strength of the material due to the special structure of the CTB interface, which resists dislocation transmission^12^. Lu et al. first discovered that submicrometer-size grained Cu composed of numerous nanoscale twins could achieve a combination of high ductility and strength^13^. It was found that limited triple junction mobility in nanotwinned structure can result in drag on the motion of the grain boundaries connected during grain growth^14^ and atomic diffusion^9^, conferring better thermal stability and electromigration resistance to nanotwinned copper (nt-Cu). These results suggest that the significant improvement in reliability is realized by nanotwinning in Cu.

To date, it has been shown that the exploration of the nanotwinned structure in FCC metals has opened up new avenues of research and applications. Recent studies have studied the use of electrodeposition and sputtering methods to produce a high density of nanotwins in face-cubic centered (FCC) metals. Lu et al. has produced Cu films with a high density of nanoscale growth twins by electroplating with a current density that is about 10 times greater than usual^8^. Liao et al. has successfully fabricated Cu nanowires (NWs) with the twinning structure by pulsed-current deposition at − 2 °C; these NWs exhibit a highly (111) crystallographic texture and an extremely small twin width of as small as 6.5 nm^15^. Hsiao et al. has also reported the formation of highly (111)-oriented Cu grains with densely packed nanotwins by direct-current electroplating with a rapid stirring rate^16^. Zhang et al. have synthesized bulk copper foils with twin spacing of 5 nm via magnetron sputtering process and developed a corresponding model that accounts for the formation of nanoscale twins during the sputtering process by manipulating the parameters (deposition rate and substrate temperature) and material properties (stacking fault energy) to control twin densities during deposition^17,18^. Xu et al. has proposed an innovative nanotwin formation mechanism in Cu thin films by stress/strain relaxation in pulse electrodeposition, in which highly strained Cu undergoes recrystallization and grain growth to relieve stress and form more energetically stable nanotwins^19^. In nanotwinned Cu fabricated by multilayer deposition technology, Hodge et al. suggests that build-in stress relaxation can occur during an interruption between the depositions of successive layers^20^. Lingk and Gross also reported the presence of twins after grain growth and recrystallization, which led to stress relaxation^21^. From these results, it can be seen that stress and stress relaxation play a significant role in the formation of nanotwins. Despite numerous previous studies on nanotwinned metals, few reports have examined the enhancing effect of substrate bias in the sputtering process for the formation of nanotwinned structures in Cu film with a highly (111)-preferred orientation. Herein, the authors systematically discuss the effect of substrate bias voltage on the microstructure of the sputtered Cu films and investigate how substrate bias can facilitate the formation of (111)-oriented Cu nanotwins.

## Results

**Figure 1:**
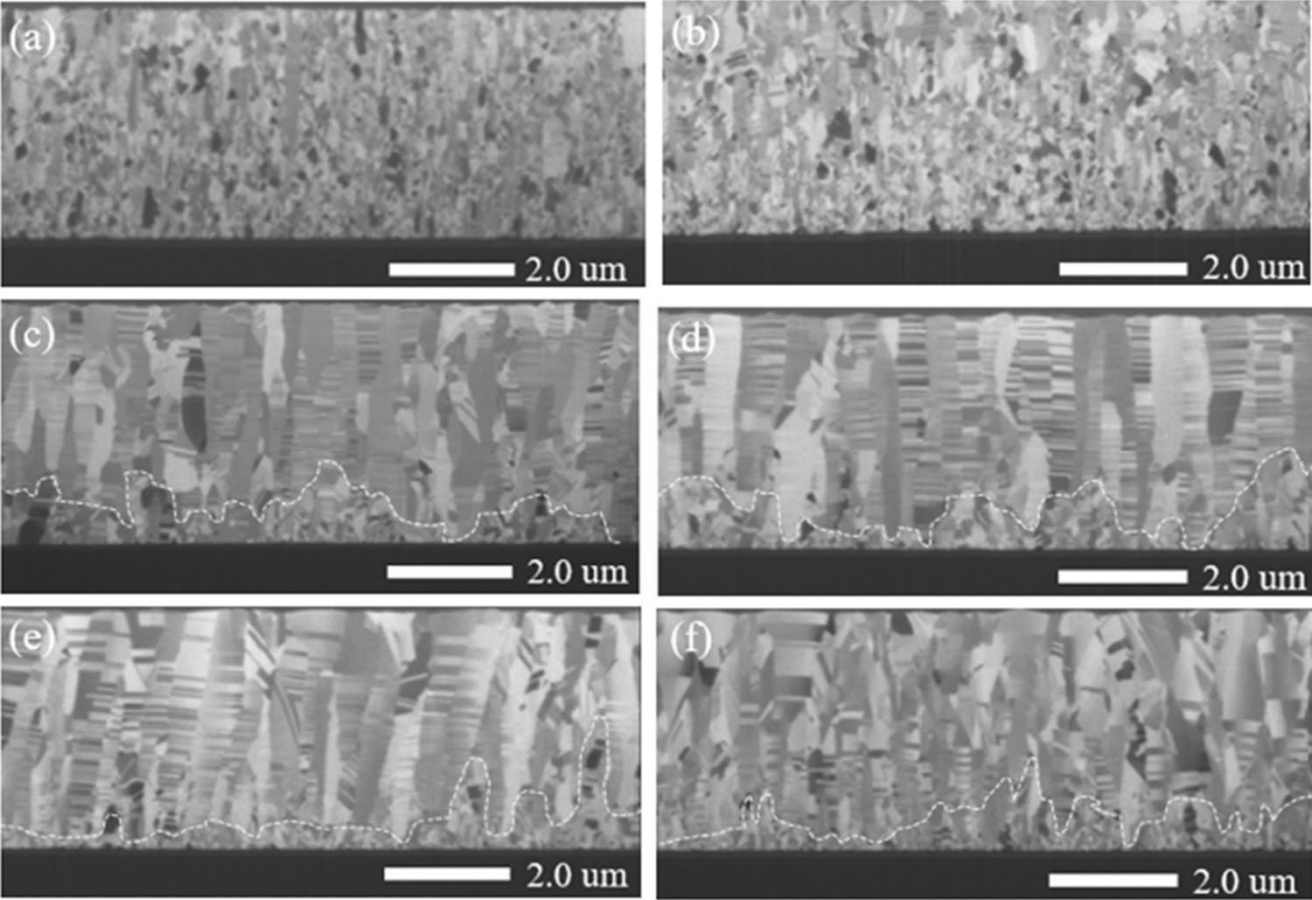
The FIB cross-sectional metallographs of the sputtered Cu films deposited with substrate biases of (**a**) 0 V, (**b**) − 50 V, (**c**) − 100 V, (**d**) − 150 V, (**e**) − 200 V and (**f**) − 250 V.

Figure 1 shows cross-sections of the sputtered Cu films on Ti-precoated SiC deposited with various substrate biases ranging from 0 to − 250 V. Obvious differences in the crystal structures can be distinguished by FIB ion images. In the Cu films deposited with substrate bias of 0 V and − 50 V, the polycrystalline Cu grains were evenly distributed over the thin film, and no Cu nanotwins were observed within the cross-section. Under the influence of a negative bias voltage, the Cu grains were found to transform to slender structured grains, and a higher proportion of Cu nanotwins formed with substrate bias of − 100 V and − 150 V, as shown in Fig. 1c and d, respectively. The biased Cu thin film was composed of random grains, marked with dashed lines and known as the transition layer, with nanotwinned columnar grains above them. These columnar grains comprised densely-packed nanotwins stacked on each other in the growth direction. When the substrate bias voltage was − 200 V and − 250 V, the nanotwin density decreased, as shown in Fig. 1e and f, respectively. Apparently, the substrate bias had significant influences on the microstructure of the Cu films and had an optimal value, as reported by Wu et al., Lai et al., and Sha et al.^22–24^.

**Figure 2:**
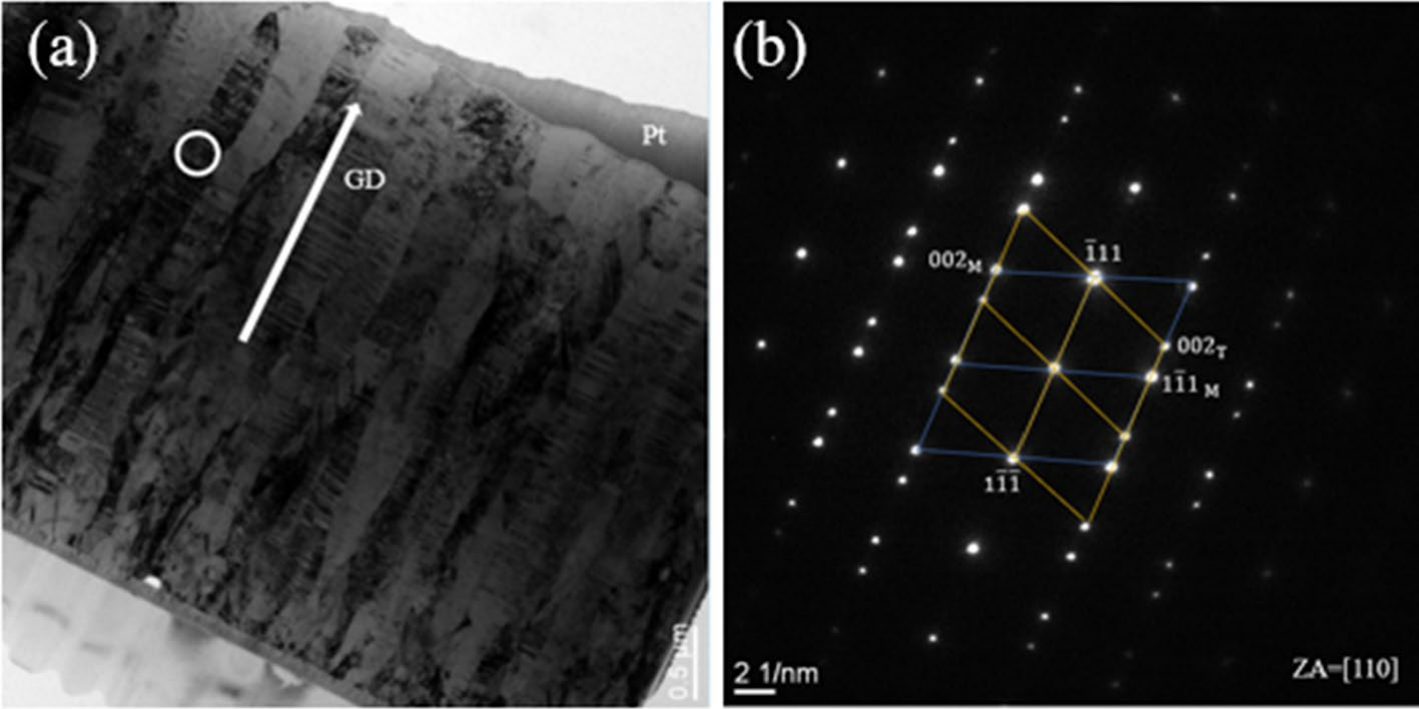
(**a**) Cross-sectional bright field TEM image of sputtered Cu nanotwins and (**b**) selected area electron diffraction (SAD) pattern obtained from the white circle in (**a**).

**Figure 3:**
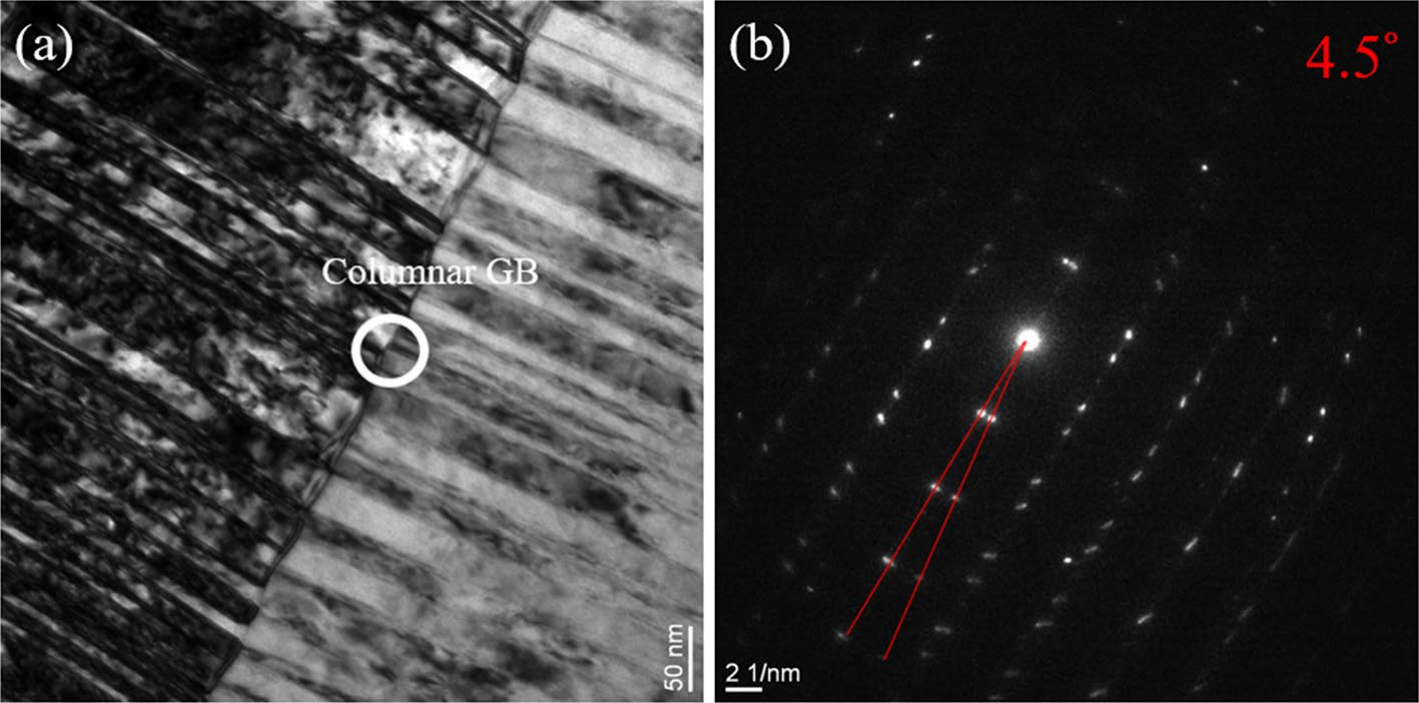
(**a**) Cross-sectional bright field TEM image of area between columnar grains. (**b**) Diffraction pattern obtained from the area in the white circle in (**a**) showing that the (111)-oriented planes across densely-packed nanotwinned columnar grains were misaligned by 4.5°.

In addition to FIB imaging, the microstructure were further analyzed by transmission electron microscopy (TEM). Cross-sectional TEM bright field images of the nanotwinned Cu film deposited with − 150 V bias are shown in Figs. 2 and 3. A high-density nanotwinned structure was observed, and the growth direction of the Cu nanotwins was perpendicular to the SiC substrates, as illustrated by the white arrow in Fig. 2a. The selected area diffraction (SAD) pattern taken along the [110] zone axis (Fig. 2b) revealed that the columnar grains circled in Fig. 2a presented two sets of distinguished diffraction spots of matrix (M) and twins (T), which were symmetrical along the $(1\overline{1 }\overline{1 }\text{)/(}\overline{1 }\text{11)}$ co-plane, demonstrating the characteristic of a twin structure. In addition, the bright field image in Fig. 3a revealed two columnar grain structures composed of numerous densely packed Cu nanotwins piled up on each other along the growth direction. The diffraction pattern in Fig. 3b obtained from the area in the white circle in Fig. 3a indicated that the (111)-oriented planes across the densely-packed nanotwinned columnar grains were misaligned by 4.5°.

**Figure 4:**
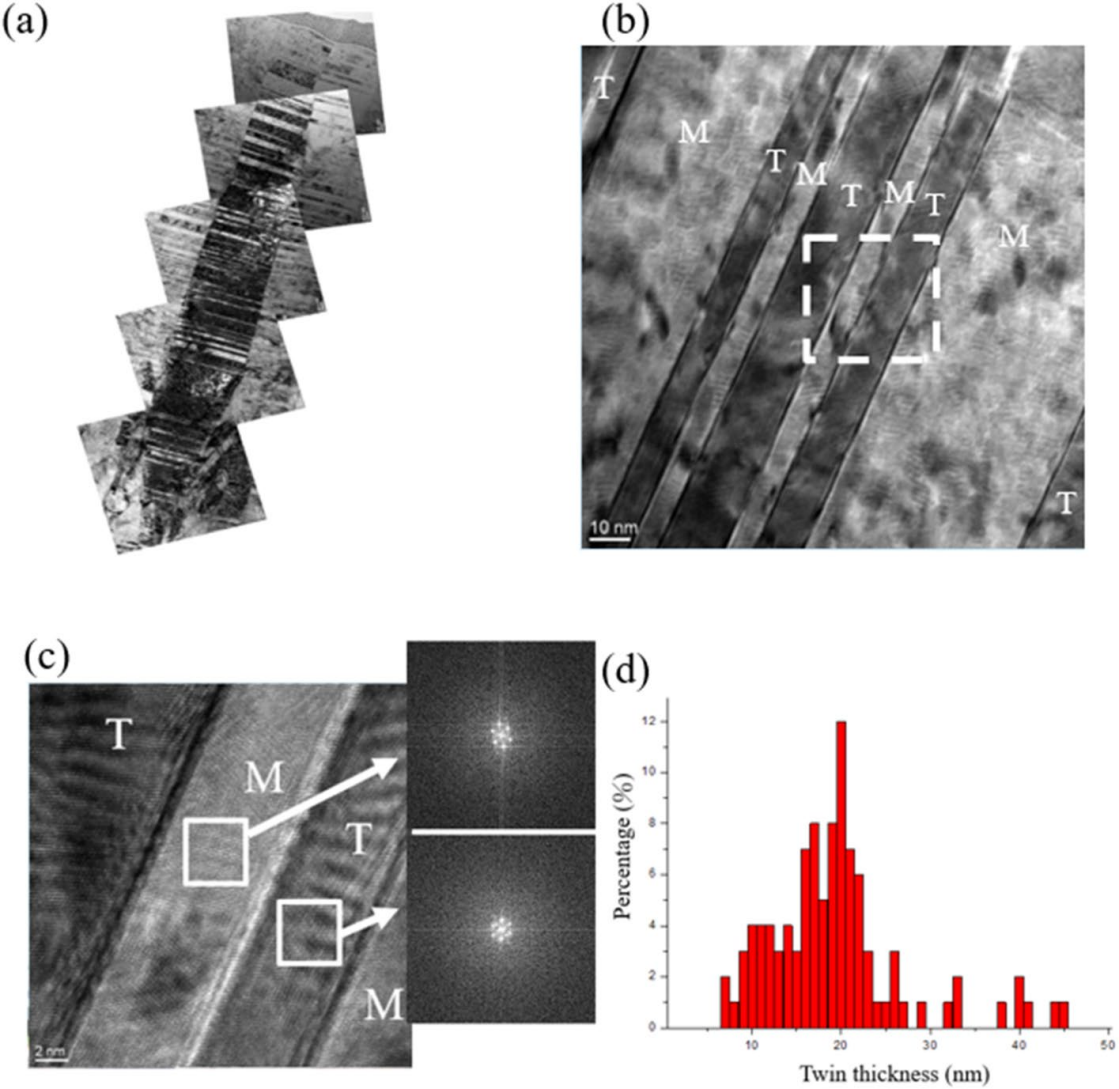
Cross-sectional TEM micrographs of as-deposited Cu nanotwinned film deposited with − 150 V bias: (**a**) bright-field TEM image of columnar Cu nanotwin (**b**) high resolution (HR) TEM image of nanotwinned structure (**c**) high magnification HRTEM image of nanotwinned structure from the white frame in (**b**) with different fast Fourier transformation (FFT) pattern of twin and matrix (**d**) distribution of twin thicknesses in the highly (111)-oriented Cu film.

The distribution of nanotwins and matrix in columnar grains can be observed clearly in the bright-field TEM image of an integral nanotwinned columnar grain shown in Fig. 4a. In addition, densely-stacked nanotwins were clearly observed along the columnar grains. Figure 4b and c present a high resolution (HR) TEM image and a Fast Fourier Transform (FFT) pattern of twin and matrix. The results showed that nanotwins and the matrix were stacked alternately. The histogram distribution of the nanotwin thicknesses is shown in Fig. 4d. Most nanotwins were distributed between 10 and 25 nm and the average twin thickness was 19.4 nm. The above measurements of the nanotwin thickness were calculated from several TEM observations of at least 200 nanotwins.

**Figure 5:**
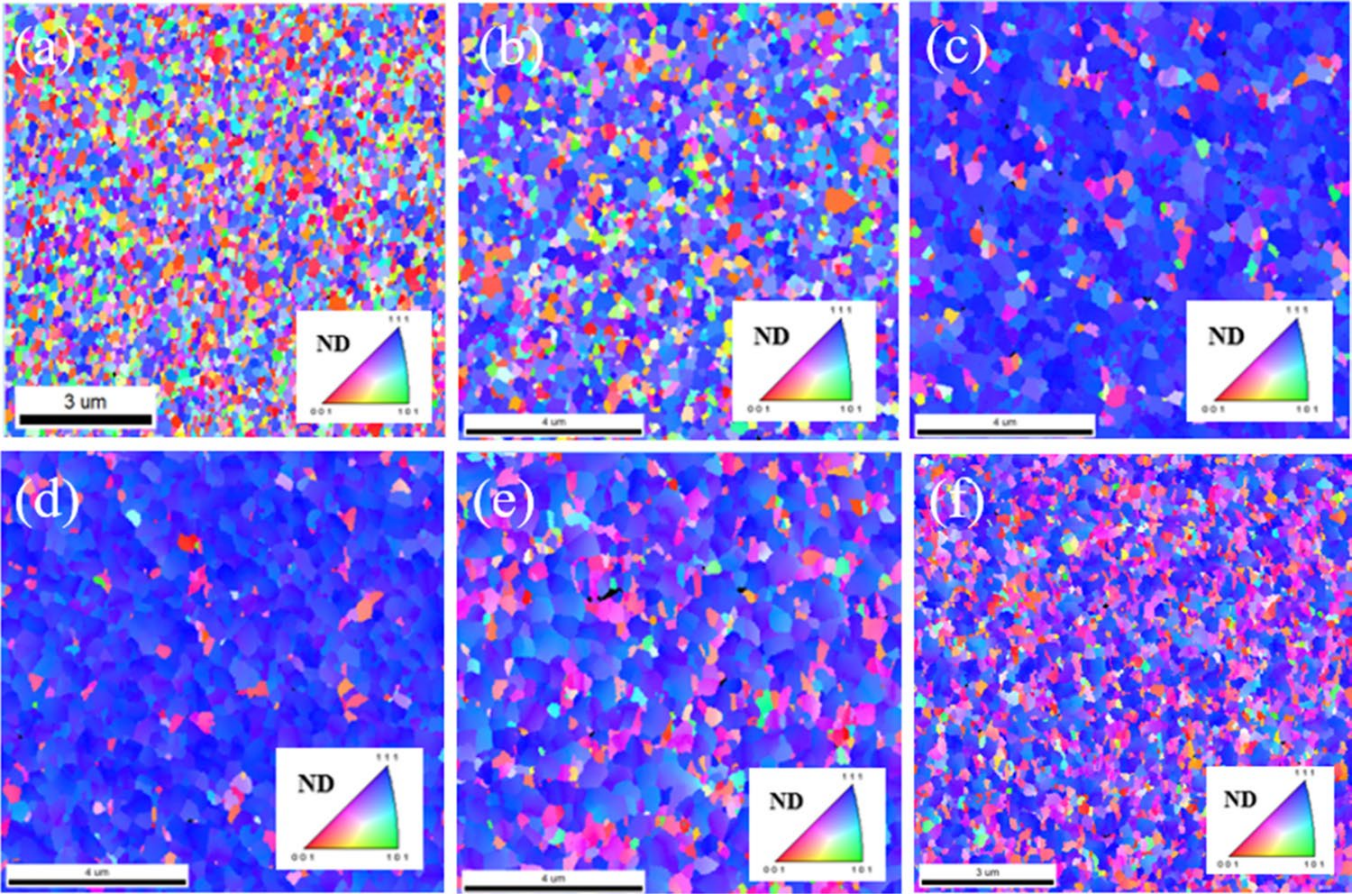
Plan-view EBSD inverse pole figure (IPF) maps of sputtered Cu films deposited with substrate biases of (**a**) 0 V, (**b**) − 50 V, (**c**) − 100 V, (**d**) − 150 V, (**e**) − 200 V and (**f**) − 250 V.

**Figure 6:**
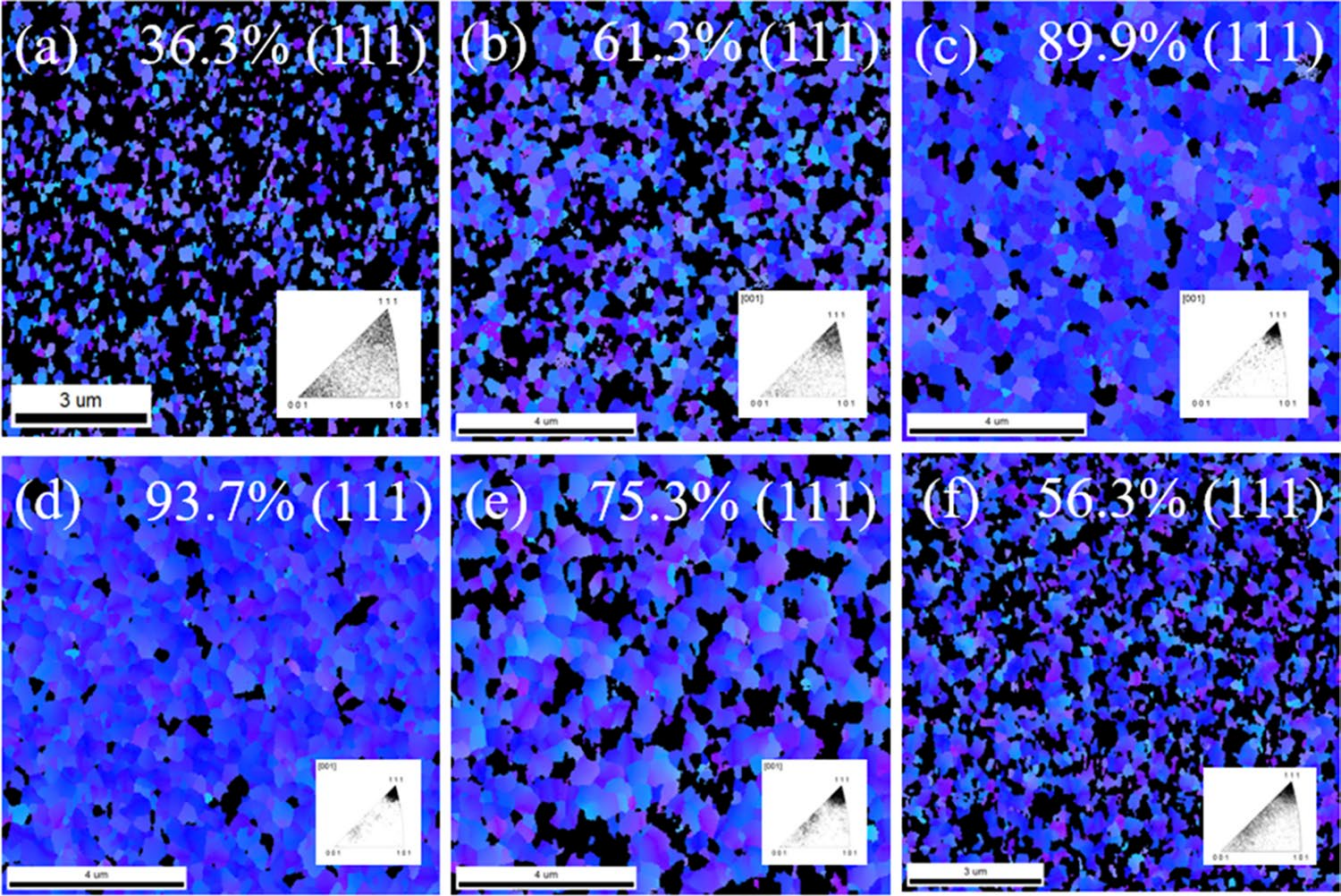
Plane-view EBSD inverse pole figure (IPF) maps of sputtered Cu films deposited with substrate biases of (**a**) 0 V, (**b**) − 50 V, (**c**) − 100 V, (**d**) − 150 V, (**e**) − 200 V and (**f**) − 250 V showing (111)-orientation.

For subsequent bonding applications, the surfaces of nanotwinned Cu films have two important criteria: the proportion of (111)-orientation and the surface roughness. EBSD measurements were performed to further characterize the grain orientations of the Cu thin films. Plane view EBSD IPF images of sputtered Cu films deposited with different substrate biases are shown in Fig. 5. Cu grains with misorientation angles exceeding 15° relative to the (111)-direction are indicated by the dark region in Fig. 6. The results showed that Cu film deposited without substrate bias consisted of randomly-oriented grains, and only 36.3% had (111) orientation. Application of the proper substrate bias substantially increased the proportion of (111)-orientation to 93.4%.

**Figure 7:**
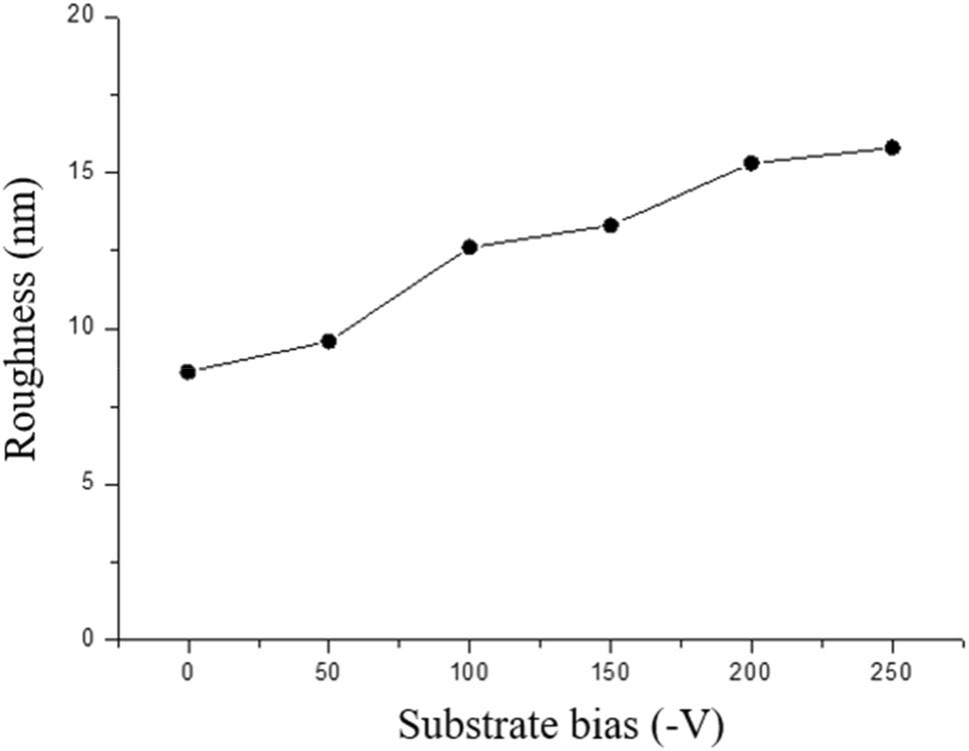
Surface roughness of sputtered Cu films deposited with various substrate biases.

In fact, the highly (111)-oriented nanotwins formed on the biased Cu film surface are expected to facilitate low-temperature direct metal bonding due to the high surface diffusivity and the low oxidation rate of that particular plane. The surface roughness of the Cu films deposited with various substrate biases were determined by an atomic force microscope (AFM), as shown in Fig. 7. The results indicated that the surface roughness of the Cu film was proportional to the value of the substrate bias voltage.

**Figure 8:**
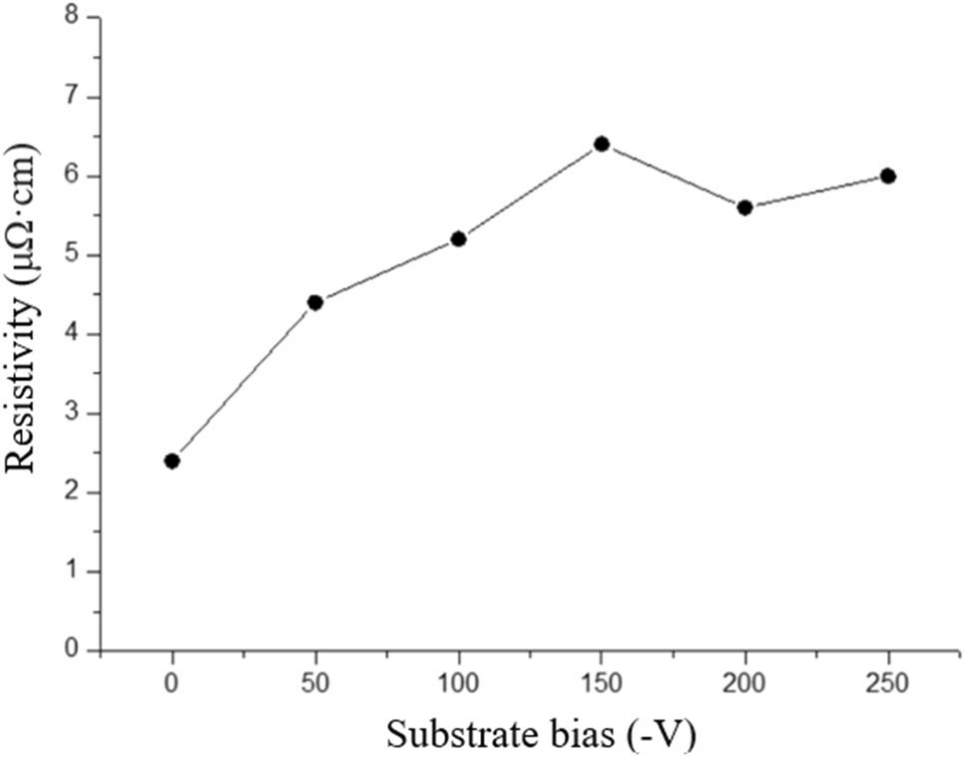
Resistivity of the Cu nanotwinned films sputtered with bias voltages of 0 V to − 250 V.

**Figure 9:**
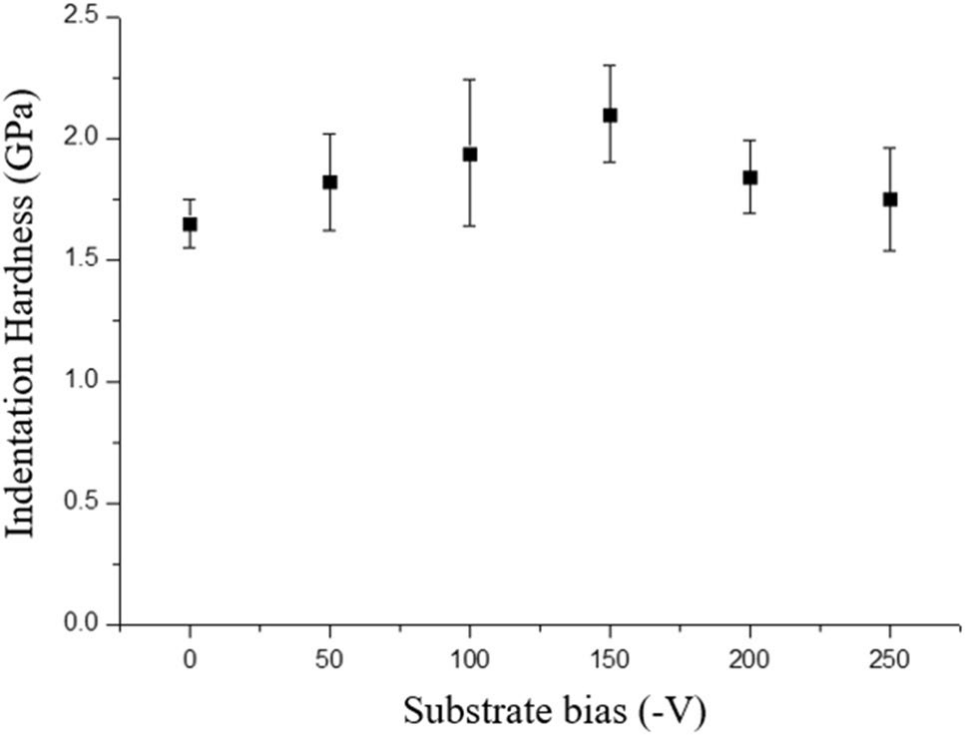
Indentation hardness of the Cu films sputtered with bias voltages of 0 V to − 250 V.

The electrical resistivity and indentation hardness of the copper nanotwinned films sputtered with different substrate biases are shown in Figs. 8 and 9. The data are also summarized in Table 1. As shown in Fig. 8 and Table 1, the resistivity of the copper nanotwinned films sputtered at various substrate biases varied. The resistivity of the Cu film increased from 2.4 to 6.4 μΩ cm when the applied substrate bias was increased from 0 to − 150 V. When the applied substrate bias exceeded − 150 V, the resistivity dropped. This trend can be explained by the variation in nanotwin density and the ion bombardment effect.

**Table 1:** Information of Cu nanotwinned films sputtered on SiC chips with various substrate biases.

Substrate bias (− V)	Microstructure	(111) preferred orientation (%)	Roughness (nm)	Resistivity (μΩ cm)	Average hardness (GPa)
0	Nanocrystalline grain	36.3	8.6	2.4	1.65
50	Nanocrystalline grain	61.3	9.5	4.4	1.8
100	High density nanotwins	89.9	12.6	5.2	1.9
150	High density nanotwins	93.7	13.3	6.4	2.1
200	Low density nanotwins	75.3	15.3	5.6	1.75
250	Low density nanotwins	56.3	15.8	6	1.7

For the mechanical property, a nanoindenter was used to measure the indentation hardness of the copper nanotwinned films deposited with different substrate voltages, as shown in Fig. 9 and Table 1. In the nanoindentation test, substrate effect and surface roughness must be considered. To avoid the substrate and surface roughness effects, the indentation depth should not exceed 10% of the overall film thickness and the surface roughness should be less than 20% of the indentation depth. In this experiment, the overall film thickness of the Cu film was about 4 um and the surface roughness was between 8.6 and 15.8 nm; thus, the indentation depth was fixed at 200 nm. As shown in Fig. 9, the average indentation hardness of the Cu film increased from 1.65 to 2.1 GPa when the applied substrate bias was increased from 0 to − 150 V. The highest value of indentation hardness was measured to be 2.3 GPa in the film deposited with − 150 V substrate bias. The existence of large numbers of CTBs may act as a strong obstacle for dislocation slip and contribute to the strength of the Cu nanotwinned films^25^.

**Figure 10:**
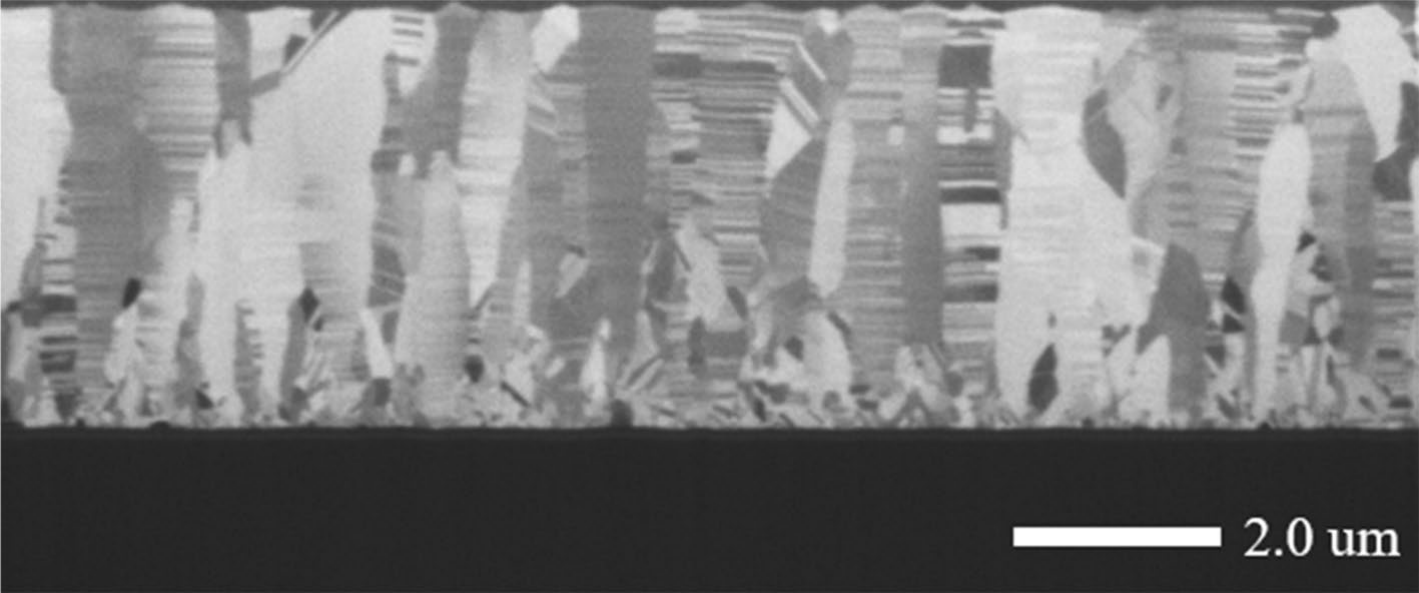
The FIB cross-sectional metallographs of the sputtered Cu films deposited with substrate bias of − 150 V on Si substrate.

For comparison, copper nanotwinned films were also sputtered on conventional Si chips with various substrate biases. As can be observed in Fig. 10, the high density nanotwins also appeared in the Cu thin film deposited on the Si chip with an optimized bias of − 150 V. This result indicates that the method of fabricating copper nanotwins by applying substrate bias during sputtering is not limited to silicon carbide substrates but can also be adapted for different substrates, which can allow wider application.

## Discussion

Nanotwinned films have been attracting considerable attention because of their outstanding mechanical properties^4–6^, good conductivity^7,8^, satisfactory electromigration resistance and superior thermal stability^9–13^, which make it feasible for them to be applied in a wide range of engineering fields. Therefore, further exploration to promote the design of nanotwinned materials has become a major focus of research. In previous studies, nanotwinned Cu had been successfully fabricated by electroplating under conditions of high current density^8^, low temperature^15^, rapid stirring rate^16^, and pulsed time^18^ to facilitate the nanotwin formation. Here, we fabricated highly (111)-oriented Cu with a high density of nanotwins by magnetron sputtering with negative substrate bias of 150 V to discuss the effect of bias on the microstructure and structural properties of the Cu films.

The microstructure zone model in evaporation was first reported by Movchan and Demchishin^26^. They found that the microstructure of an evaporated coating of Ti, W and ZrO could be represented as a function of T/${\mathrm{T}}_{m}$, where T and ${\mathrm{T}}_{m}$ are the deposition and melting temperature, respectively, as three zones, each with its own characteristic structure and physical properties. The zone model was subsequently extended to magnetron sputtering by adding an axis to account for the effect of the Ar working pressure and Zone T between Zone 1 and Zone 2 by Thornton^27^. Zone T presents a fibrous texture without voided boundaries due to the increased adatom mobility from energetic particle bombardment. In the sputtering process, the major parameters that influence the film microstructure are the ratio of T/${\mathrm{T}}_{m}$ and particle bombardment. The cross-sectional FIB ion images in Fig. 1d show that a dense columnar grain structure appeared in Zone T with ion bombardment. When the substrate bias voltage was applied during deposition, Ar ions were attracted to bombard the films, transferring the energy to the adatoms on the substrate. The strain energy became larger during the bombardment and tended to be relieved through nanotwinning. From the thermodynamic point of view, the sum of the interfacial energies including grain boundaries and twin boundaries will be reduced by twinning because the excess energy for coherent TBs is much smaller than that for high-angle GBs. In addition, stresses induced by the impingement of the Ar ions on the film surface are also crucial for the twin formation within the Cu films. Similar results were observed in Cu films fabricated by electroplating bombarded by 5 keV of Ar ions at low temperature^28^, indicating that the thermal spike cascades induced by ion bombardment cause the generation and motivation of twinning partial dislocations.

The preferred orientation can be adjusted by varying the substrate bias and realized by the formation of nanotwins induced by ion bombardment, which increase the strain energy. CTBs have an ordered and symmetrical boundary structure, and they typically exhibit much lower energy than conventional high-angle GBs. In Cu, for example, CTBs, ITBs, and high-angle GBs have energies of 24–39 mJ/m^2^, 590–714 mJ/m^2^, and 625–710 mJ/m^2^, respectively^29,30^. To reduce the sum of the interfacial energies, high density CTBs will be formed. In addition, CTBs have a strong tendency to align perpendicular to the growth direction, so columnar grains with a strong {111} texture are common in fcc metals on most substrates. Similar results have been found in metals with appropriately low stacking fault energies, such as Cu^7,8,17,20^ and Ag^22,23^.

Cu bonding is of great interest due to its potential to replace solder joints in advanced packing technologies. Typical solder reflows usually occur at temperatures of about 250 °C, so lowering the direct bonding temperature down to 250 °C has become a key challenge. Since the essential process in direct bonding is known to be surface diffusion, the (111) orientation of Cu is known to have the highest surface diffusion coefficient, which is 3 to 4 orders of magnitude more rapid than those of other planes, implying that the bonding temperature can be lowered further on Cu (111) than on the other planes^31^. Here, we expected that the highly (111)-oriented Cu film would facilitate low temperature direct bonding because of the high surface diffusivity and low surface roughness. The results of the biased Cu film revealed that the entire surface was colored in blue, indicating that over 90% of the film surface was covered by (111) Cu nanotwins. In contrast, the microstructure of the unbiased Cu film was random, without any preferred orientation. An oriented (111) texture of extremely high degree, up to 93.7%, provided rapid paths for diffusion and enabled direct bonding to be carried out at a fast pace.

In low temperature direct bonding, another important factor is surface roughness. As shown in Fig. 7. The results indicated that the surface roughness of the Cu film was proportional to the value of the substrate bias voltage. The reason was that the increase in substrate bias voltage accelerated the ion bombardment energy, and surface defects formed by the higher energy of incident ions roughened the surface. When the sputtered species impinged onto the surface, not only energy transference between the sputtered species and film atoms but also collisions occurred. On the other hand, excessive ion bombardment can create defects at higher rates than can be repaired by the enhanced mobility of surface species. Therefore, higher substrate bias would induce a rougher surface.

To further investigate the effect of substrate bias on nanotwin formation in sputter deposited Cu films, we therefore examined the cross-sectional film structure in detail using TEM analysis. The bright field TEM and HRTEM images revealed epitaxial nanotwinned Cu films, with an average twin spacing of 19.4 nm. In addition, (111)-oriented planes across the densely-packed nanotwinned columnar grains were misaligned by 4.5°, as confirmed by the selected area electron diffraction (SAD) pattern in Fig. 3b. Grain boundaries with misorientation of less than ~ 10° are generally considered to be low-angle grain boundaries (LAGB), the interfacial energies of which are markedly lower than those of general HAGBs. From the thermodynamics perspective, HAGBs are seen as the major driving force in grain growth because of their high energy relative to the energy of LAGBs and twin boundaries. In addition, coherent twin boundaries carry much lower stored energy than high-angle grain boundaries and hence have a greatly reduced driving force for the coarsening of twins^12^. Anderoglu et al. also reported the thermal stability of Cu thin film with a high density of nanoscale growth twins by annealing at up to 800 °C^10^. That thin film exhibited better thermal stability than did monolithic nanocrystals with high-angle grain boundaries. Combining numerous twin boundaries and LAGBs, the Cu nanotwinned films in this study can be expected to exhibit better thermal stability and mechanical properties contributed by these stable structures. On the other hand, twin spacing is another important factor that affects the mechanical properties of Cu nanotwins. Similar to the concept of nanograining, nanotwinning has a critical twin spacing for achieving the maximum mechanical strength. Proposed by Lu et al., the strength of nanotwinned Cu increased with decreasing twin spacing and eventually reached a maximum value at around 15 nm^8^. In this study, the indentation hardness of Cu film with twin spacing of about 19.4 nm was measured to be 2.3 GPa. In this case, the twin lamella thickness plays a dominant role in controlling film hardness^10^. It is known that twin and grain boundaries can act as strong obstacles to dislocation motion and that the twin lamella thickness plays a dominant role in controlling film hardness. Therefore, the distinct increase in the hardness of the film is ascribed to the addition of the substrate bias, which facilitated the formation of the nanotwinned structure. In addition to the mechanical properties, the electrical properties were also affected by structural changes caused by the applied substrate bias. As shown in Fig. 8, the resistivity of Cu films slightly increased with increases in the applied substrate bias from 0 to − 150 V. A higher substrate bias not only benefits twinning but also results in higher energetic impingement of Ar^+^ ions, which introduces local atomic disorder and thus promotes scattering sites for the electrical carriers. These ions have sufficient energy to produce radiation-induced defects such as dislocation loops and point defect clusters, which increase resistivity. When the bias voltage is higher than − 200 V, a decline in film resistivity is observed. This phenomenon is attributed to the decrease in twin density because fewer CTBs are present to cause electron scattering.

## Conclusion

In the present work, the microstructure of Cu films was shown to be strongly affected by substrate bias during sputtering deposition. The overall results provided good agreement for the enhancement effect of applied substrate bias on the formation of the highly (111)-oriented Cu nanotwins. Ion bombardment caused by the substrate bias during growth was apparently effective in densifying the deposition and contributed to the formation of densely-packed Cu nanotwins within the columnar structures. FIB and EBSD analyses showed numerous Cu nanotwins stacked on one another along the growth direction, and up to 93.7% of the grains formed on the film surface were (111)-oriented. The electrical and mechanical properties of the nanotwinned Cu film were influenced by the structural changes resulting from the applied substrate bias. As also supported by other experimental studies in the literature, the technique of ion bombardment during deposition to induce the formation of nanotwins can be extended to other FCC metals with low stacking fault energy.

## Methods

### Preparation of Cu films

In this study, thin films were deposited with a DC magnetron sputtering system (JUBSUN TECH, SGS-500) with a base pressure < 5 Torr × 10^−6^ (6 Pa × 10^−4^). Argon gas, maintained at 5 mTorr (0.67 Pa), was used as a working gas in the deposition process. Prior to each film deposition, all targets were pre-sputtered for 10 min to minimize contamination. The SiC and Si substrates were properly cleaned with acetone, ethanol and deionized water subsequently to promote adhesion during deposition. Before deposition, the substrate was pre-sputtered by argon discharge at a bias of − 500 V for 5 min to remove the surface oxide layer. After bombardment, the Cu films were sputtered with a sputter rate of 1.12 nm/s and sputtering power of 300 W. Ti was pre-coated on the substrate as an adhesive layer to promote adhesion between the Cu and the substrate. Different negative substrate biases were applied to induce Ar ion bombardment to the substrate surface during deposition. After deposition, the samples were left in the chamber for cooling to room temperature.

### Cu film analysis

The samples were then further analyzed by a focused ion beam (FIB, Hitachi NX2000) to observe the microstructure of the thin films and prepare specimens for the subsequent transmission electron microscopy (TEM, FEI Tecnai G2 F20). For further microscopic observation of the thin films, TEM was employed to identify the twin thicknesses, Cu nanotwin arrangement and misalignment angle between the Cu columnar grains. EBSD analysis was performed to examine the grain orientations of the surfaces of the Cu films. The crystallographic information from EBSD patterns was obtained by Orientation Imaging Microscopy (OIM) software (TSL, USA). The roughness (Ra) of the thin film surfaces was measured with an atomic force microscope (AFM, Burker Dimension Edge). For the electrical property, the film resistivity was identified with a four-point probe (Napson RT-70). For the mechanical property, the indentation hardness was determined by nanoindenter (Hysitron TI 980 TriboIndenter).

## Data Availability

The datasets generated during and/or analysed during the current study are available from the corresponding author on reasonable request.
